# Increased incidence of melanoma in children and adolescents in Finland in 1990–2014: nationwide re-evaluation of histopathological characteristics

**DOI:** 10.1080/07853890.2022.2026001

**Published:** 2022-01-17

**Authors:** Emma K. Rousi, Roope A. Kallionpää, Roosa E. Kallionpää, Susanna M. Juteau, Lauri A. I. Talve, Micaela M. Hernberg, Pia P. Vihinen, Veli-Matti Kähäri, Ilkka O. Koskivuo

**Affiliations:** a Department of Plastic and General Surgery, Turku University Hospital and University of Turku, Turku, Finland; b Institute of Biomedicine, University of Turku, University of Turku, Turku, Finland; c Auria Biobank, Turku University Hospital and University of Turku, Turku, Finland; d Central Laboratory of Pathology, Helsinki University Hospital, Helsinki, Finland; e Department of Pathology, Turku University Hospital, Turku, Finland; f Comprehensive Cancer Center, Helsinki University Hospital, Helsinki, Finland; g Tyks Cancer Centre and FICAN West, University of Turku and Turku University Hospital, Turku, Finland; h Department of Dermatology, University of Turku and Turku University Hospital, Turku, Finland; i FICAN West Cancer Centre Laboratory, University of Turku and Turku University Hospital, Turku, Finland

**Keywords:** Melanoma, incidence, spitzoid melanoma, malignant Spitz tumour, children, adolescents

## Abstract

**Background:**

Changes in the incidence of melanoma in children and adolescents have been reported in Europe and in the USA in the recent decades.

**Aims:**

The aim of this study was to examine the incidence of paediatric and adolescent melanomas in Finland in 1990-2014, and the associated clinical and histopathological characteristics to reveal temporal trends, such as changes in diagnostic sensitivity of Spitzoid melanomas.

**Methods:**

Information on 122 patients diagnosed with cutaneous melanoma at 0-19 years of age in Finland in 1990-2014 were retrieved from the Finnish Cancer Registry. 73 primary melanoma archival samples were re-evaluated by two dermatopathologists to allow comparability over time.

**Results:**

A 5.6% annual increase was observed in the incidence of melanoma among children and adolescents during the study period. Fifty-six tumours were confirmed as malignant melanomas in the re-evaluation. After correction for tumour misclassification in the Cancer Registry, the age-adjusted annual incidence was estimated to have increased from 1.4/1 000 000 in 1990-1994 to 5.8/1 000 000 in 2010-2014. The change in incidence was most prominent among adolescents and in Spitzoid melanoma subtype. Melanomas diagnosed 1990-2002 and 2003-2014 did not differ in terms of their clinicopathological characteristics or prognosis (hazard ratio for melanoma-related death 1.53, 95% CI 0.30 to 7.88). Spitzoid melanomas were diagnosed at a younger age, were of higher stage and had higher Clark level than other melanomas, yet the hazard ratio for death was 0.52 (95% CI 0.10 to 2.58) for Spitzoid versus other melanomas.

**Conclusions:**

The incidence of cutaneous melanoma has clearly increased among the young in Finland, especially among adolescents. No evidence for overdiagnosis of Spitzoid melanomas as the underlying cause of the increased incidence was observed.

**Key message**
A nationwide retrospective re-evaluation of the cutaneous melanomas recorded in the Finnish Cancer Registry among patients aged 0–19 years in Finland in 1990–2014 revealed an approximately 4-fold increase in the incidence. The increase in the incidence was most prominent among adolescents and in the Spitzoid melanoma subtype. Our results contrast those reported in other countries, where the incidence of melanoma among adolescents has declined.

## Introduction

An increase in the incidence of melanoma in children and adolescents has been reported in Europe and in the USA during the recent decades, especially among adolescents, yet the reasons underlying this change are unclear [1–6]. In Sweden, the incidence of melanoma among adolescents increased since the 1970s and decreased in 1998–2002 [4,7]. In the United States, the incidence was observed to increase until 2004, and thereafter decline especially among adolescents [8–10]. Similar trend has also been seen in the Netherlands [3].

Changes in protective behaviour towards sun exposure, as well as restrictions in the use of tanning beds in the United States have been suggested to have decreased the incidence in the last decade [4,7,11]. Another possible cause for the changes in the melanoma incidence could be related to the evolution of histopathological diagnostic criteria for Spitzoid melanoma in the recent decades [12–14]. This melanoma subtype, often found in children and adolescents, has diverse mutational spectrum and can be difficult to distinguish histopathologically from its less aggressive mimic, atypical Spitz tumour, yet several features associated with malignant behaviour have been described [12,13]. During the last two decades, the awareness of Spitzoid melanomas and their malignant behaviour has increased, which may have resulted in overdiagnosis and, consequently, an apparent increase in their incidence [15–19].

In our previous studies, we examined the primary tumour characteristics, treatment and clinical course of cutaneous melanoma in cohorts of Finnish paediatric and adolescent melanoma patients [5,6]. In the present study, we aim at investigating the age-adjusted incidence of melanoma in children and adolescents in Finland in 1990–2014 based on Finnish Cancer Registry (FCR) data together with centralised review of melanoma archival samples. The second aim of the study is to investigate whether there is evidence of overdiagnosis of melanomas in children and adolescents, and Spitzoid melanomas in particular, which could affect the apparent melanoma incidence among the young.

## Materials and methods

Information on all patients (*n* = 122) diagnosed with cutaneous melanoma <20 years of age in 1990–2014 were retrieved from the Finnish Cancer Registry (FCR) database and re-evaluated as previously described [5]. Tissue samples were available for re-evaluation by two dermatopathologists with experience on the diagnosis of paediatric melanoma (SJ and LT) in 73 cases, and the diagnosis of malignant melanoma based on the 4^th^ edition of the WHO histopathological diagnostic criteria was made by our pathologists in 56 cases (Figure S1) [13]. Samples that were non-malignant according to both dermatopathologists were considered as originally misclassified in the FCR data. Immunohistochemical labelling for BRAF V600E, ALK and PD-L1 were analysed as previously described [5]. The follow-up ended at last information on patient status (2014–2019), or death.

The study was approved by the Ethics Committee of the Hospital District of Southwest Finland, Helsinki University Hospital, National Institute for Health and Welfare and Valvira National Supervisory Authority for Welfare and Health (Licenses ETMK:140/1803/2014, HUS/83/2019, THL/552/5.05.00/2016, Valvira: 6479/06.01.03.01/2016).

### Statistical analysis

Age-adjusted melanoma incidence and its confidence intervals were estimated using the method described by Fay and Feuer [20]. The Finnish population in 2014 was used as the standard population for age-adjustment, and the numbers of individuals aged 0–19 years were obtained from Statistics Finland in 1-year strata for years 1990–2014. Annual incidence per 1 000 000 individuals was visualised as point estimates and 95% confidence intervals. To highlight any trends, the data were smoothed using cubic splines. Annual percent change and changes of incidence trends were computed using the Joinpoint software (version 4.8.0.1) allowing for 0–2 joinpoints. In the Joinpoint analysis, one year (1994) had to be omitted since no melanomas were observed.

The total observed incidence was calculated using the melanomas reported in the FCR. In addition, another analysis was stratified by age group (0–10 and 11–19 years) to reveal potential differences between smaller children and adolescents. Since not all the tissue samples of the tumours reported in the FCR were available for re-evaluation, the true incidence of melanoma was estimated based on the proportions of confirmed melanomas in the available samples. To obtain incidence estimates corrected for misclassification, the study period was divided into five-year intervals, and the proportion of confirmed melanomas out of all re-evaluated samples was calculated for each interval and applied to the corresponding annual numbers of melanomas reported in the FCR. In addition, an analysis stratified by the presence of Spitzoid features was performed among the confirmed melanomas.

To examine time-related differences, the study period was divided into sub-periods of 1990–2002 and 2003–2014. Melanoma characteristics were compared between the two sub-periods among the 56 confirmed melanomas. In addition, confirmed melanomas with Spitzoid features were compared with the other melanomas. The Chi-squared test was used to compare melanoma location, tumour type, sentinel lymph node biopsy (SLNB) result and the extent of tumour-infiltrating lymphocytes (TILs). Fisher’s exact test was utilized for comparisons of sex, metastatic disease, deaths, ulceration, and BRAF V600E, PD-L1 and ALK expression. Mann–Whitney U-test was applied to age at diagnosis, Clark level, Breslow thickness, density of mitoses and tumour stage. Survival probability was estimated using the Kaplan–Meier method, and hazard ratio (HR) for death was computed using the Cox proportional hazards model. Statistical significance was determined at the two-sided 5% level.

All analyses except the Joinpoint analysis were performed using the R software for statistical computing (version 4.0.0) and packages dsrTest (version 0.2.1) and survival (version 3.1-12).

## Results

**Figure 1.**
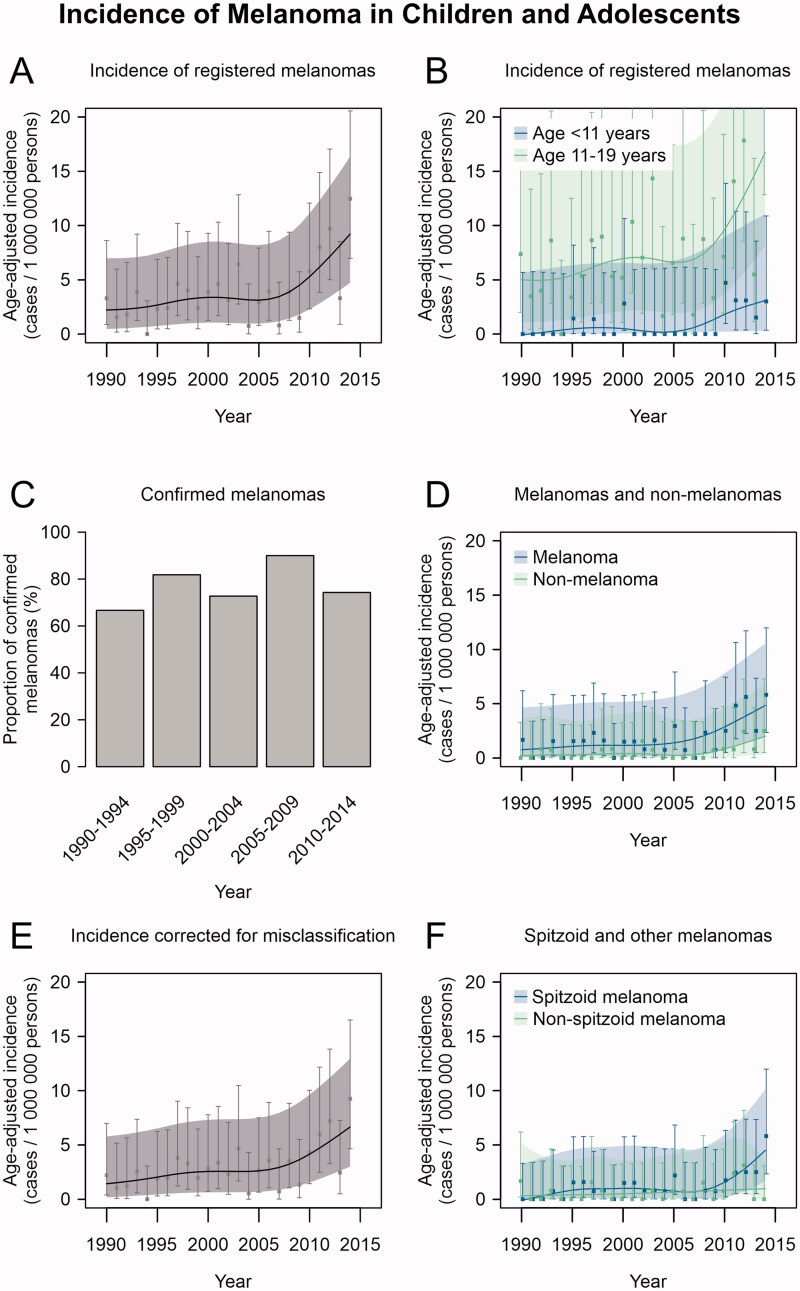
Trends of melanoma incidence in Finland in 1990–2014. (A) Incidence of melanoma cases reported in the Finnish Cancer Registry (FCR). (B) Incidence of melanoma cases reported in the FCR by age group. (C) The proportion of confirmed melanomas out of all the tumours available for re-evaluation in 5-year intervals of diagnosis date. (D) Incidence of confirmed melanomas and non-melanomas among the cases available for re-evaluation (E) Total population incidence of melanoma corrected for misclassification. (F) Incidence of Spitzoid and other melanomas among the cases available for re-evaluation. All incidence estimates have been age-adjusted relative to the Finnish population structure in the year 2014. Age-adjusted point estimates (squares) and their 95% confidence intervals (whiskers) are shown along with estimates and confidence intervals that have been smoothed using cubic splines (continuous lines and shading, respectively).

Analysis of the 122 patients recorded in the FCR revealed a statistically significant increasing trend with annual percent change of 5.6% (95% CI 2.7 to 8.6) in the age-adjusted melanoma incidence (Figure 1(A)). A change in the trend could be observed in 2009 (95% CI 2001 to 2012) if one joinpoint was introduced into the analysis, but the model did not significantly differ from the analysis without any joinpoints (*p* = 0.082). When the analysis was stratified by age, the increase in incidence was visually most prominent among adolescents aged 11–19 years, and the increase in melanoma incidence was modest among children <11 years (Figure 1(B)).

The proportion of tumours confirmed as malignant melanomas out of all the re-evaluated tumours remained stable over the study period, with the proportion of melanomas ranging from 67% to 90% in five-year periods (Figure 1I; *p* = 0.835). Consequently, increasing trends were observed in the incidence of both melanomas and non-melanoma tumours (Figure 1(D)), yet formal analysis was not possible because of the low number of cases per year. Considering the proportion of misclassified tumours in the FCR data during each five-year period, the estimated true mean annual incidence of melanoma among children and adolescents in Finland increased from 1.4/1 000 000 in 1990–1994 to 5.8/1 000 000 in 2010–2014 (Figure 1(E)). The incidence of Spitzoid melanomas seemed to increase in the most recent years, while no such increase was seen in other melanomas (Figure 1(F)).

### Comparison of primary melanoma characteristics in 1990–2002 and 2003–2014

The characteristics of the 56 melanomas reviewed for this study were compared between the time periods 1990–2002 and 2003–2014 (Table 1). SLNB had been performed in 68% (*n* = 26) of the more recent cases, while SLNB had only been performed in one patient (5.6%) before 2003. SLNB was introduced in Finland in the early 2000s. Consequently, the tumours diagnosed in 2003–2014 were of slightly higher stage than tumours diagnosed earlier (*p* = 0.100). There were no differences in Clark level, Breslow thickness, ulceration, or mitotic rate between the two time periods (Table 1). Melanoma-related deaths appeared to be more frequent in the pre-2003 period. However, patients diagnosed 1990–2002 had a median follow-up time of 18.0 years (range 2.6 to 25.4) and those diagnosed 2003–2014 had a median follow-up time of 5.3 years (range 0.58 to 14.8). There was no significant difference in the relative risk of melanoma-related death before 2003 compared to the period after 2003 (HR 1.53, 95% CI 0.30 to 7.88, *p* = 0.611).

**Table 1.** Characteristics of melanomas diagnosed in 1990–2002 and 2003–2014.

	1990–2002	2003–2014	*p* Value
Cases (*n*)	18	38	
Sex, *n* (%)			
Males	9 (50.0)	18 (47.4)	1.000
Females	9 (50.0)	20 (52.6)	
Age at diagnosis, mean (SD) (years)	16.7 (3.2)	14.9 (3.8)	0.089
Metastasis during follow-up, *n* (%)	3 (16.7)	4 (10.5)	0.669
Melanoma-related deaths during follow-up, *n* (%)	3 (16.7)	3 (7.9)	0.374
Melanoma location, *n* (%)			
Head and neck	2 (11.1)	10 (26.3)	0.464
Trunk	4 (22.2)	10 (26.3)	
Upper extremity	2 (11.1)	4 (10.5)	
Lower extremity	10 (55.6)	13 (34.2)	
Not available	0 (0.0)	1 (2.6)	
Clark level, *n* (%)			
1	1 (5.6)	2 (5.3)	0.207
2	4 (22.2)	2 (5.3)	
3	6 (33.3)	15 (39.5)	
4	7 (38.9)	17 (44.7)	
5	0 (0.0)	2 (5.3)	
Not available	0 (0.0)	0 (0.0)	
Breslow thickness			
Median (range) (mm)	1.1 (0.2 to 5.0)	1.6 (0.6 to 15.3)	0.237
Not available, n (%)	1 (5.6)	2 (5.3)	
Ulceration, *n* (%)			
Yes	2 (11.1)	7 (18.4)	0.703
No	16 (88.9)	31 (81.6)	
Not available	0 (0.0)	0 (0.0)	
Tumour-infiltrating lymphocytes, *n* (%)			
Low	10 (55.6)	18 (47.4)	0.851
Moderate	7 (38.9)	16 (42.1)	
High	0 (0.0)	1 (2.6)	
Not available	1 (5.6)	3 (7.9)	
Mitoses / mm^2^			
Median (range)	0 (0 to 18)	1 (0 to 12)	0.541
<6 mitoses / mm^2^, *n* (%)	14 (77.8)	31 (81.6)	0.670
≥6 mitoses / mm^2^, *n* (%)	3 (16.7)	4 (10.5)	
Not available, *n* (%)	1 (5.6)	3 (7.9)	
BRAF V600E expression, *n* (%)			
Positive	9 (50.0)	17 (44.7)	0.771
Negative	8 (44.4)	20 (52.6)	
Not available	1 (5.6)	1 (2.6)	
PD-L1 expression, *n* (%)			
Positive	0 (0.0)	1 (2.6)	1.000
Negative	17 (94.4)	36 (94.7)	
Not available	1 (5.6)	1 (2.6)	
ALK expression, *n* (%)			
Positive	1 (5.6)	4 (10.5)	1.000
Negative	16 (88.9)	33 (86.8)	
Not available	1 (5.6)	1 (2.6)	
Melanoma type, *n* (%)			
Superficial spreading	5 (27.8)	7 (18.4)	0.525
Nodular	0 (0.0)	4 (10.5)	
Spitzoid	12 (66.7)	25 (65.8)	
*In situ*	1 (5.6)	2 (5.3)	
Not available	0 (0.0)	0 (0.0)	
Tumour stage, *n* (%)			
0	1 (5.6)	2 (5.3)	0.100
I	10 (55.6)	16 (42.1)	
II	6 (33.3)	5 (13.2)	
III	1 (5.6)	14 (36.8)	
IV	0 (0.0)	1 (2.6)	
Not available	0 (0.0)	0 (0.0)	
Sentinel lymph node biopsy, *n* (%)			
Positive	0 (0.0)	12 (31.6)	<.001
Negative	1 (5.6)	14 (36.8)	
Not performed	17 (94.4)	11 (28.9)	
Not available	0 (0.0)	1 (2.6)	

The *p*-values represent the difference between the two time periods and were computed among the cases where the information was available.

The proportions of the melanomas occurring in head and neck, trunk, upper and lower extremity did not differ between the two time periods (Table 1). Also, melanoma location did not differ by age group (0–10 and 11–19) or by sex between the time periods. Moreover, there was no significant difference in the proportions of melanomas located in presumably sun-exposed (head and neck, upper extremities) and non-sun-exposed (trunk, lower extremities) sites (*p* = 0.241). Despite the increase in the incidence of Spitzoid melanomas during the last five years of the study period (Figure 1(F)), the proportion of Spitzoid melanomas did not differ between 1990–2002 and 2003–2014 (Table 1).

### Characteristics of the Spitzoid melanomas

**Figure 2.**
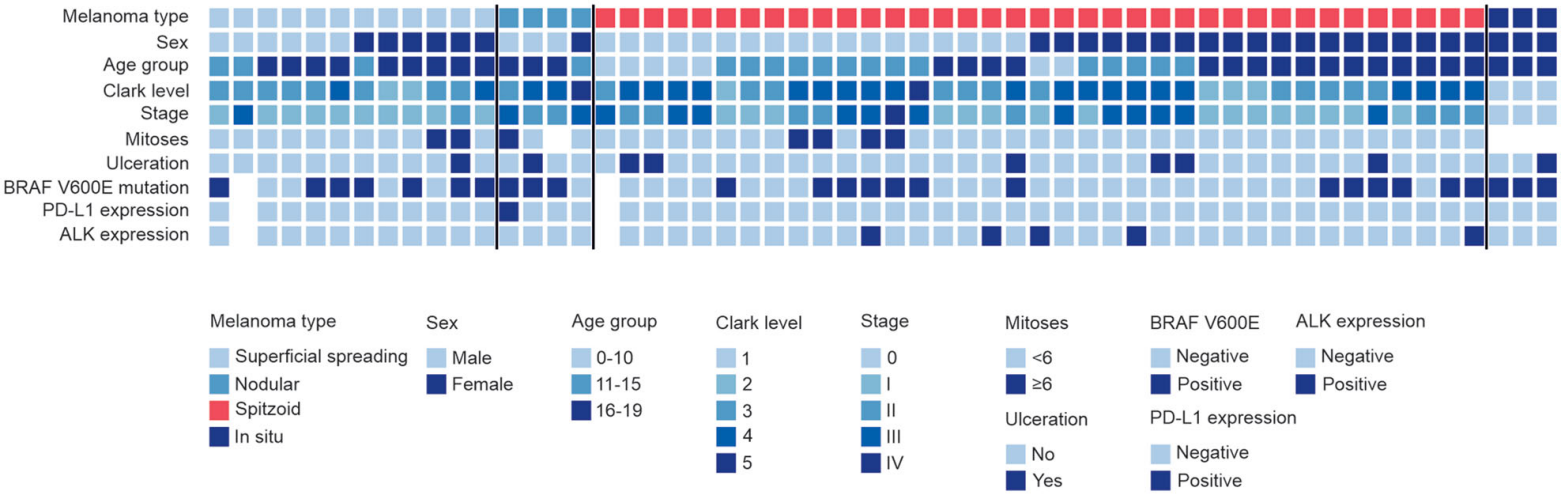
Primary melanoma characteristics. Individual-level characteristics of the melanomas re-evaluated in the study. Spitzoid melanomas are shown with red colour on the melanoma type row.

There were 37 Spitzoid melanomas among the 56 melanoma samples, representing 66% of the melanomas (Table 2; Figure 2). Spitzoid features were also observed in 9/17 (53%) originally misclassified benign or borderline samples re-evaluated in this study (Table S1), and in 12/16 cases with discordant diagnoses from the two dermatopathologists (Figure S1).

**Table 2.** Characteristics of Spitzoid and other melanomas.

	Spitzoid melanoma	Other melanoma types	*p* Value
Cases (*n*)	37	19	
Sex, *n* (%)			
Males	18 (48.6)	9 (47.4)	1.000
Females	19 (51.4)	10 (52.6)	
Age at diagnosis, mean (SD) (years)	14.5 (4.0)	17.4 (2.0)	0.006
Year of diagnosis, mean (SD)	2006.5 (6.9)	2004.5 (7.9)	0.270
Metastasis during follow-up, *n* (%)	3 (8.1)	4 (21.1)	0.212
Melanoma-related deaths during follow-up, *n* (%)	3 (8.1)	3 (15.8)	0.397
Melanoma location, *n* (%)			
Head and neck	7 (18.9)	5 (26.3)	0.530
Trunk	8 (21.6)	6 (31.6)	
Upper extremity	4 (10.8)	2 (10.5)	
Lower extremity	18 (48.6)	5 (26.3)	
Not available	0 (0.0)	1 (5.3)	
Clark level, *n* (%)			
1	0 (0.0)	3 (15.8)	0.030
2	4 (10.8)	2 (10.5)	
3	12 (32.4)	9 (47.4)	
4	20 (54.1)	4 (21.1)	
5	1 (2.7)	1 (5.3)	
Not available	0 (0.0)	0 (0.0)	
Breslow thickness			
Median (range) (mm)	1.7 (0.2 to 6.0)	1.2 (0.2 to 15.3)	0.455
Not available, *n* (%)	0 (0.0)	3 (15.8)	
Ulceration, *n* (%)			
Yes	6 (16.2)	3 (15.8)	1.000
No	31 (83.8)	16 (84.2)	
Not available	0 (0.0)	0 (0.0)	
Tumour-infiltrating lymphocytes, *n* (%)			
Low	21 (56.8)	7 (36.8)	0.303
Moderate	16 (43.2)	7 (36.8)	
High	0 (0.0)	1 (5.3)	
Not available	0 (0.0)	4 (21.1)	
Mitoses / mm^2^			
Median (range)	1 (0 to 12)	1 (0 to 18)	1.000
<6 mitoses / mm^2^, *n* (%)	33 (89.2)	12 (63.2)	0.397
≥6 mitoses / mm^2^, *n* (%)	4 (10.8)	3 (15.8)	
Not available, *n* (%)	0 (0.0)	4 (21.1)	
BRAF V600E expression, *n* (%)			
Positive	13 (35.1)	13 (68.4)	0.020
Negative	23 (62.2)	5 (26.3)	
Not available	1 (2.7)	1 (5.3)	
PD-L1 expression, *n* (%)			
Positive	0 (0.0)	1 (5.3)	0.333
Negative	36 (97.3)	17 (89.5)	
Not available	1 (2.7)	1 (5.3)	
ALK expression, *n* (%)			
Positive	5 (13.5)	0 (0.0)	0.157
Negative	31 (83.8)	18 (94.7)	
Not available	1 (2.7)	1 (5.3)	
Tumour stage, *n* (%)			
0	0 (0.0)	3 (15.8)	0.025
I	16 (43.2)	10 (52.6)	
II	8 (21.6)	3 (15.8)	
III	12 (32.4)	3 (15.8)	
IV	1 (2.7)	0 (0.0)	
Not available	0 (0.0)	0 (0.0)	
Sentinel lymph node biopsy, *n* (%)			
Positive	11 (29.7)	1 (5.3)	0.143
Negative	9 (24.3)	6 (31.6)	
Not performed	17 (45.9)	11 (57.9)	
Not available	0 (0.0)	1 (5.3)	

The *p*-values represent the difference between the two groups and were computed in the cases where the information was available.

Spitzoid melanomas had been diagnosed at an average age of 14.5 years, while the mean age at the diagnosis of the other melanoma types was 17.4 years (*p* = 0.006; Table 2). Forty-nine percent of the Spitzoid melanomas were located in lower extremities, while this was the case in only 26% of the other melanomas (*p* = 0.153). Spitzoid melanomas were of higher stage and had higher Clark level compared to other melanomas, whereas there was no significant difference between the groups in Breslow thickness, ulceration, mitotic rate, or TILs (Table 2). The melanoma-specific Kaplan–Meier estimate of five-year survival after the diagnosis of Spitzoid melanoma was 94.5% (95% CI 87.4% to 100%), and the relative risk of death after melanoma diagnosis was not significantly different among those with Spitzoid melanoma compared to those with other melanoma types (HR 0.52, 95% CI 0.10 to 2.58). BRAF V600E -positivity was less frequent in Spitzoid melanomas compared to the other melanoma types (35.1% vs. 68.4%), while all five tumours positive for ALK were Spitzoid melanomas. SLNB was performed in 27/56 patients, out of which the result was positive in 11/20 (55%) patients with Spitzoid melanomas and 1/7 (14%) patients with other melanomas, but the difference was not statistically significant (*p* = 0.091).

## Discussion

After correction of the age-adjusted melanoma incidence for originally misclassified tumours registered in the FCR, a substantial increase from 1.4/1 000 000 to 5.8/1 000 000 was observed among children and adolescents during the study period. This increase could either be due to a true change of melanoma incidence or changes in diagnostic criteria and register coverage. The incidence rate of paediatric melanoma observed in Finland in 2010–2014 is comparable with the rates reported in the USA and in the Netherlands [1–3,8]. Differences between countries may be related to cultural or social characteristics, quality and coverage of melanoma registration or, for example, health education efforts aimed to reduce UV exposure.

True changes in melanoma incidence may be caused by increased UV exposure in more recent years. Living at northern latitudes is associated with a lower risk of melanoma also in the adolescents, and the northern location of Finland likely limits the domestic sun exposure [21,22]. However, recreational travel has increased remarkably during the study period and may have contributed to the UV exposure. Finns made a total of 1.97 million overnight trips abroad in 1991, while the number was 5.88 million in 2014 [23].

The classification of Spitzoid melanomas has significantly improved in recent decades [13,14]. As a result, Spitzoid tumours previously not regarded as melanomas and therefore not registered in the FCR could have been classified as Spitzoid melanomas under the revised criteria in recent years. This may have improved register coverage and caused apparent increase in the incidence of melanoma in children and adolescents without a true change in incidence. However, the proportion of Spitzoid melanomas out of all reviewed melanomas did not differ between the sub-periods of the study period. The lack of difference between the two sub-periods may be due to the choice of cut point, because an increasing trend of Spitzoid melanoma incidence was seen in Figure 1(F). Nevertheless, Spitzoid melanomas may have been classified as malignant melanomas based on alarming histological features already before the introduction of clear diagnostic criteria. If Spitzoid melanomas had been misdiagnosed as benign tumours before the establishment of specific criteria, some of those melanomas would likely have metastasised and led to death without treatment. Since the FCR collects information on cancers also from death certificates, initially misdiagnosed melanomas would have been eventually registered. However, none of the melanomas registered among children and adolescents in Finland in 1990–2014 were based on death certificate information only.

The histopathological diagnosis of Spitzoid tumours has been challenging even for experts [16,24]. This was also observed in our study, as the Cohen’s kappa for inter-rater agreement of 0.50 (Figure S1) indicates that drawing the line between Spitzoid melanoma and a non-malignant (Spitzoid) tumour is highly demanding even for experienced dermatopathologists. The tumours with discordant diagnoses were typically associated with favourable prognostic factors, such as low mitosis count, low Breslow thickness and lack of ulceration. The majority of these tumours (75%) had Spitzoid features.

The Finnish Cancer Registry data suggested some level of overdiagnosis of paediatric and adolescent melanomas, as some of the registered samples were considered non-malignant in our re-evaluation by both dermatopathologists. However, the rates of original misclassification observed in the present study did not significantly differ between the different 5-year periods in 1990–2014. Because of the lack of significant differences in the prognosis or key prognostic factors between melanomas diagnosed pre- and post-2003, or between Spitzoid and other melanomas, the time between tumour formation and diagnosis seems to have remained similar throughout the study period. The present results therefore provide no convincing evidence to suggest that the increase in melanoma incidence would be caused by increased awareness of paediatric melanoma or overdiagnosis of Spitzoid melanomas in more recent years.

Spitzoid melanomas in children often metastasise into regional lymph nodes without worsening the prognosis [5,6,11]. Therefore, completion lymph node dissection following a positive SLNB is now recommended in children and adolescents only when clinically or radiographically positive lymph nodes are present, similarly to adults [11,25]. Despite the stage migration through the introduction of SLNB, there was no evidence of patients diagnosed before the SLNB era having a worse prognosis. Since Spitzoid melanomas exhibit many evolutionary pathways, some of the tumours are likely to behave more aggressively than others [18,19,26]. Accordingly, there is need for reliable biomarkers to aid the diagnosis and treatment of these rare tumours. The diagnosis of Spitzoid melanoma is still based on histopathology and there is a risk for overtreatment of children and adolescents with this malignancy.

Our conclusions are limited by the lower number of patients compared with studies performed in larger countries [3,10,27]. As a result, any subgroups are small, which hampers our ability to study associations between melanoma characteristics and age or calendar year. Moreover, calculating the annual percent change of incidence was not possible within subgroups or using only melanomas confirmed in the re-evaluation due to the small number of cases per year. Nevertheless, the material represents the total Finnish population over a 25-year period. While not all melanomas diagnosed in Finland during the study period were available for re-evaluation, the subset of 73 tumours is likely representative of the total of 122 tumours recorded in the FCR. As in the case of other rare diseases, meta-analyses combining material from several populations are required to detect subtle associations. The Finnish population is also genetically less heterogenic compared to, for example, the USA [28]. However, our nationwide and comprehensive data allow reliable estimation of the incidence and characteristics of melanoma among children and adolescents in Finland. The revisions in diagnostic criteria highlight the value of re-evaluating archival samples according to a single version of diagnostic criteria to allow comparable results between different decades, as done in the present study. Centralised re-evaluation of tissue samples provides a more reliable way of studying incidence trends than solely relying on register-based information, which provides a large number of patients, but excludes detailed comparisons of tumour characteristics [3,4,10,27]. While the re-evaluation led to exclusion of some samples and therefore decreased the sample size, the conclusions are likely more reliable than with a slightly larger sample without re-evaluation. Studies using curated material are therefore valuable in highlighting potential clinicopathological trends in cancer epidemiology.

## Conclusions

There has been an approximately 4-fold increase in the age-adjusted incidence of melanoma among children and adolescents in Finland in 1990–2014. The increase in incidence was most prominent among adolescents and in Spitzoid melanoma subtype. Melanoma prognosis and key prognostic factors remained similar throughout the study period.

## Supplementary Material

Supplemental Material

## Data Availability

Research data are not shared due to patient data protection regulations.
