# Supplementary material for: Increased incidence of melanoma in children and adolescents in Finland in 1990–2014: nationwide re-evaluation of histopathological characteristics
Source: Ann Med. 2022 Jan 17;54(1):244–52. doi: 10.1080/07853890.2022.2026001 (PMC8765276; doi:10.1080/07853890.2022.2026001)
Supplement: Supplemental Material [file IANN_A_2026001_SM9722.zip › Supplemental files/FigS1AoM.pptx]

## Slide 1
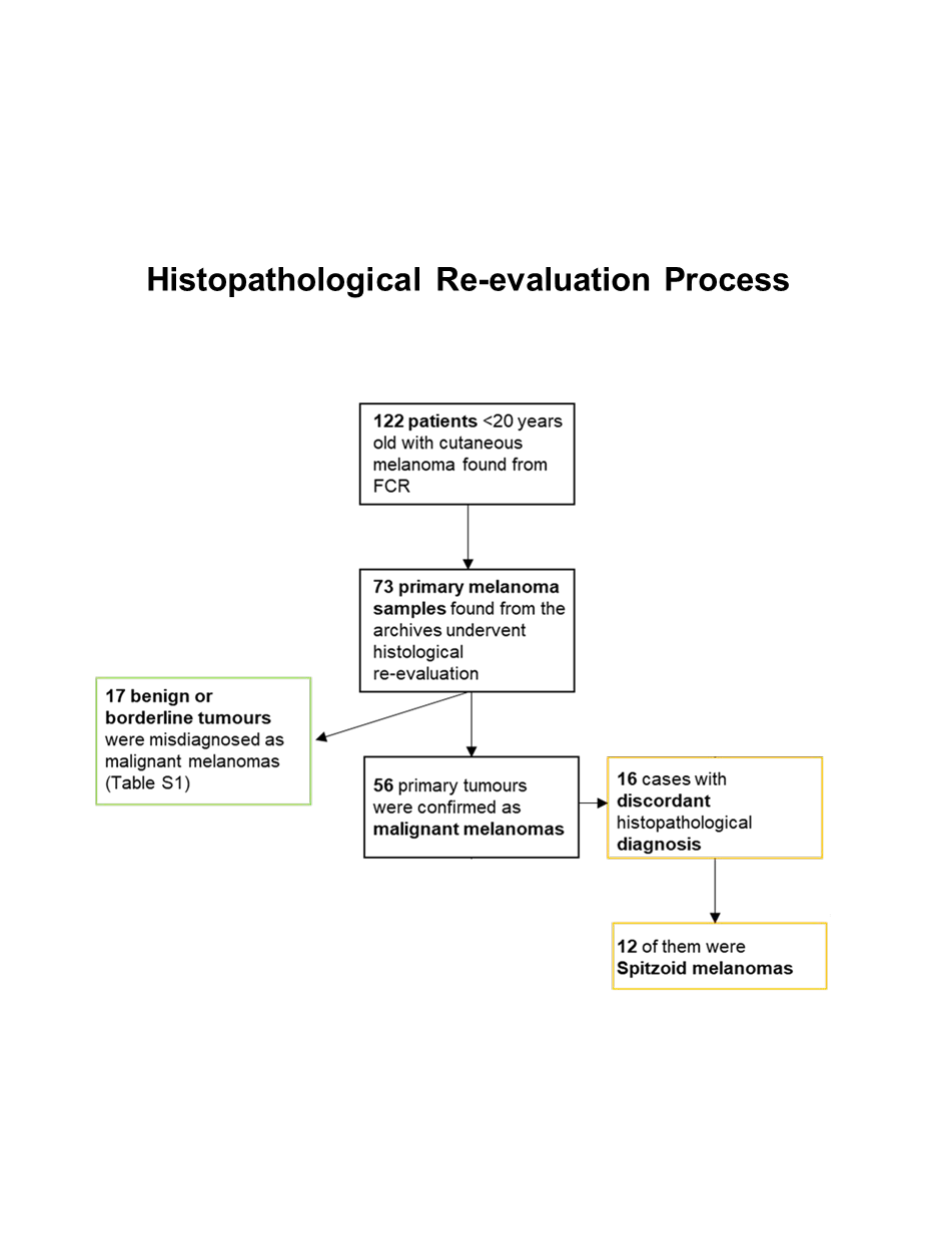

## Slide 2
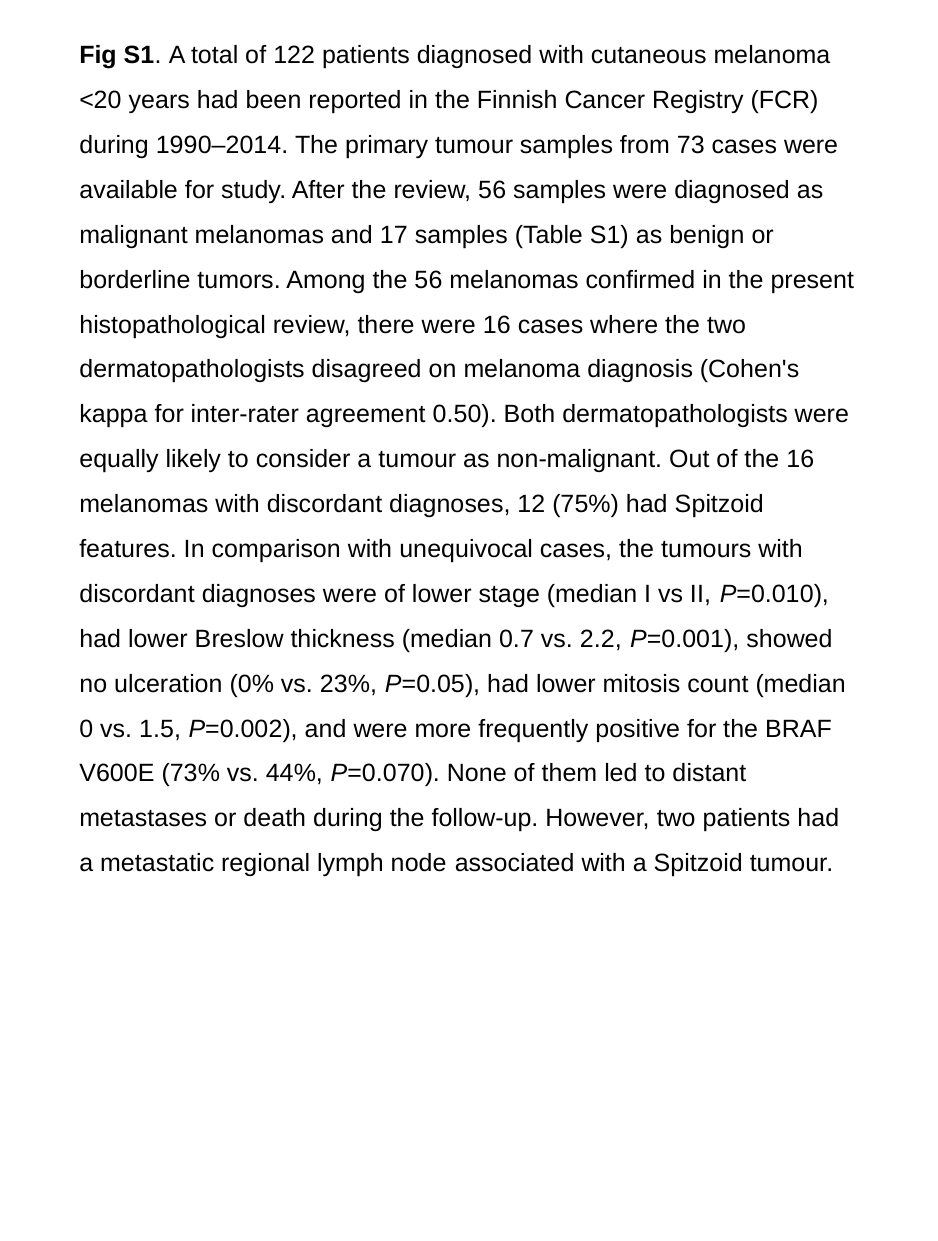

Fig S1. A total of 122 patients diagnosed with cutaneous melanoma <20 years had been reported in the Finnish Cancer Registry (FCR) during 1990–2014. The primary tumour samples from 73 cases were available for study. After the review, 56 samples were diagnosed as malignant melanomas and 17 samples (Table S1) as benign or borderline tumors. Among the 56 melanomas confirmed in the present histopathological review, there were 16 cases where the two dermatopathologists disagreed on melanoma diagnosis (Cohen's kappa for inter-rater agreement 0.50). Both dermatopathologists were equally likely to consider a tumour as non-malignant. Out of the 16 melanomas with discordant diagnoses, 12 (75%) had Spitzoid features. In comparison with unequivocal cases, the tumours with discordant diagnoses were of lower stage (median I vs II, P=0.010), had lower Breslow thickness (median 0.7 vs. 2.2, P=0.001), showed no ulceration (0% vs. 23%, P=0.05), had lower mitosis count (median 0 vs. 1.5, P=0.002), and were more frequently positive for the BRAF V600E (73% vs. 44%, P=0.070). None of them led to distant metastases or death during the follow-up. However, two patients had a metastatic regional lymph node associated with a Spitzoid tumour.
