# Supplementary material for: Increased incidence of melanoma in children and adolescents in Finland in 1990–2014: nationwide re-evaluation of histopathological characteristics
Source: Ann Med. 2022 Jan 17;54(1):244–52. doi: 10.1080/07853890.2022.2026001 (PMC8765276; doi:10.1080/07853890.2022.2026001)
Supplement: Supplemental Material [file IANN_A_2026001_SM9722.zip › Supplemental files/TableS1AoM.docx]

|  | **All** | **Males** | **Females** | **Age 0–10** | **Age 11–15** | **Age 16–19** |
| --- | --- | --- | --- | --- | --- | --- |
| **Cases (n)** | 17 | 6 | 11 | 2 | 2 | 13 |
| **Age at diagnosis (mean, SD)** | 16.5 (3.8) | 15.2 (4.6) | 17.3 (3.4) | 7.1 (0.3) | 14.4 (0.7) | 18.3 (0.9) |
| **Year of diagnosis (mean, SD)** | 2006.5 (7.6) | 2005.5 (8.5) | 2007 (7.4) | 2013 (1.4) | 2007.5 (6.4) | 2005.3 (8.0) |
| **Tumour location, n (%)** |  |  |  |  |  |  |
| Head and neck | 1 (5.9) | 0 (0.0) | 1 (9.1) | 0 (0.0) | 0 (0.0) | 1 (7.7) |
| Trunk | 7 (41.2) | 2 (33.3) | 5 (45.5) | 0 (0.0) | 0 (0.0) | 7 (53.8) |
| Upper extremity | 2 (11.8) | 0 (0.0) | 2 (18.2) | 0 (0.0) | 0 (0.0) | 2 (15.4) |
| Lower extremity | 4 (23.5) | 2 (33.3) | 2 (18.2) | 1 (50.0) | 1 (50.0) | 2 (15.4) |
| Not available | 3 (17.6) | 2 (33.3) | 1 (9.1) | 1 (50.0) | 1 (50.0) | 1 (7.7) |
| **Tumour type, n (%)** |  |  |  |  |  |  |
| Dysplastic nevus | 3 (17.6) | 2 (33.3) | 1 (9.1) | 1 (50.0) | 0 (0.0) | 2 (15.4) |
| Compound nevus | 5 (29.4) | 1 (16.7) | 4 (36.4) | 0 (0.0) | 1 (50.0) | 4 (30.8) |
| Spitz nevus | 4 (23.5) | 2 (33.3) | 2 (18.2) | 0 (0.0) | 0 (0.0) | 4 (30.8) |
| Atypical Spitz tumour | 5 (29.4) | 1 (16.7) | 4 (36.4) | 1 (50.0) | 1 (50.0) | 3 (23.1) |
| **Tumours with Spitzoid features, n (%)** | 9 (52.9) | 3 (50.0) | 6 (54.5) | 1 (50.0) | 1 (50.0) | 7 (53.8) |

***Table S1.*** *Characteristics of the benign or borderline tumours that had been registered in the Finnish Cancer Registry as malignant melanomas and were found to be non-malignant when the archival samples were re-evaluated.*
